# Risk Management Project on medication reconciliation within an acute psychiatric unit in Ireland.

**DOI:** 10.1192/j.eurpsy.2023.2183

**Published:** 2023-07-19

**Authors:** A.-M. Curtin

**Affiliations:** North Cork Mental Health Service, St Stephen’s Hospital, Glanmire, Ireland

## Abstract

**Introduction:**

Medication Reconciliation is the formal process for creating the most comprehensive and accurate list of a patient’s current medications and comparing the list to those in the patient notes and medication record. Medication Reconciliation is a time-consuming process and numerous errors can occur during the admission, inpatient stay, transfer and discharge of a patient. Errors in this process can lead to serious clinical outcomes for the patient.

**Objectives:**

The main aim for undertaking this project is to reduce the risk of medication errors during the admission process, inpatient stay, transfer, and discharge. The ultimate goal of this project is to obtain 100% compliance regarding complete medication reconciliation.

**Methods:**

Two audits were completed in an Irish Acute Psychiatric Unit in May 2021 and February 2022. Ten inpatient clinical notes and corresponding medication records were reviewed. The three stages of Medication Reconciliation were audited. Stage 1 involved collecting the data. This included reviewing all medication information sources on admission and then documenting the Best Possible Medication History. Stage 2 involved confirming the accuracy of the medication history by verifying with one or more sources (e.g. General Practioner, Community Mental Health Team, Pharmacy). Stage 3 involved comparing the Best Possible Medication History with the Precribed Medication List in the patient’s Kardex. A Medication Safety workshop was provided for all psychiatric trainees and consultants within the service and the guidelines regarding the importance of medication reconciliation were discussed.

**Results:**

**Table:** 

Results	May 2021	Feb 2022
Medication list in Initial Assessment document	90%	90%
Medication Reconciliation completed in Kardex	60%	70%
Source of Medication reconciliation documented	100%	100%

**Conclusions:**

The audit results demonstrate that there has been an improvement in medication reconciliation during the nine-month period. To obtain 100% compliance, the service needs to continue to highlight the importance of medication reconciliation practise amongst all medical staff through clinical practice, teaching sessions and regular audits.

**Disclosure of Interest:**

None Declared

